# Persistence of Bacteria within Sequestra

**Published:** 1918-03

**Authors:** 


***Persistence of Bacteria within Sequestra.*** By Major KENNETH TAYLOR, U. S. R., and MARY DAVIES, from the Laboratories of the Robert Walton Goelet Research Fund, American Red Cross Hospital, Paris, Abs. from the *Annals of Surgery,* Dec. 1916.

Because of the frequency of old suppurating fractures from war wounds, the authors investigated the bacterial flora of such wounds especially within the bone itself. Sequestra from a continuous series of secondary operations on suppurating compound fractures were examined. These varied in age from 45 to 300 days. In most cases the specimens were removed at operation about 130 days after the injury.

*Method of Examination.* — To determine the difference between the flora of the wound itself and the flora within the sequestrum, the following technique was employed. The flora of the wound was obtained by placing the sequestrum in sterile saline solution and shaking it well : the resulting emulsion was then inoculated into deep agar, upon agar plates, into anaerobic broth, into meat culture medium, and into milk. The flora of the sequestrum was obtained by removing the sequestrum from the saline emulsion, drying and flaming it in alcohol to destroy the organisms outside the bone, and then inoculating it into deep agar or meat. Later on it was removed from the culture tube, decalcified, sectioned, and studied histologically.

*Distribution of Bacteria.* — A large and varied flora was found both in the wound and in the interior of the sequestrum (streptococci, staphylococci, diphtheroid bacilli, B. proteus, B. aerogenes capsulatus, B. malignans œdematis, Hibler IX were the commonest).

Within the substance of 90 o/o of the sequestra examined histologically, bacteria were seen, usually in nests within the canals or cell spaces. It was also observed that most organisms were more commonly recovered from the sequestra than from the soft tissues. This was especially true of the anaerobes. There was an incidence of 14 o/o for the gas-forming bacilli in the wound but in the sequestra the incidence was 55 o/o. In the wound the incidence of anaerobic spores was only 7 o/o, but in the sequestra it was 42 o/o. These observations were based upon 48 consecutive cases. The authors conclude that the bone itself is the most common site for the persistence of the anaerobic flora.

Dividing the cases according to age (above and below 120 days) the authors noted a much higher incidence of anaerobes in both the wound and the sequestra of the older cases than in those of newer ones. The differences for other groups of bacteria were less marked.

The authors explain the *persistence of bacteria within the sequestra* on the ground of the mechanical protection from body fluids afforded by the dense bone structure, and they remark that leucocytes were very rarely seen within the specimens of sequestra examined.

The authors endeavored to sterilize bone sequestra *in vitro* by strong antiseptics. Small splinters of only a few millimeters in diameter were soaked for half an hour or more in alcohol 75 o/o, hydrogen peroxide, Dakin's solution, bichloride of mercury 1 : 10,000, 5 o/o carbolic acid or tincture of iodine. A good growth of organisms was nevertheless obtained after the splinter was broken and resown into a favorable medium.

Another condition in the sequestra favoring the persistence of organisms may be the combining of the acids, produced during the growth of the bacteria, with the insoluble bone salts. Thus by-products that tend to limit their growth are removed, as is the case when they are grown *in vitro* in chalk tubes.

*Erosive Action of Bacteria on Sequestra.* — Tests *in vitro* on dried pieces of compact beef bone were made to ascertain the erosive action of bacteria on sequestra. Pieces of bone were incubated in a meat culture of various organisms for a period of 10 days. It was observed that the Hibler bacillus caused the greatest loss in weight (8 o/o). The gas bacillus and B. malignans œdematis caused a loss of 6 o/o. Streptococcus and B. proteus caused a loss of 3 1/2 o/o and staphyloccocus only 2 o/o, no more than that of the uninoculated control. The authors think this loss cannot be accounted for entirely by the digestion of protein matter in the bone, for the same pieces of bone were used for as many as three successive experiments and the decrease in weight continued.

*Erosive Action of Certain Organic Acids on Sequestra.* — Since organisms produce acids which may act as solvents upon the bone substance, experiments were carried out to ascertain the relative solvent action of 1 o/o solutions of acetic, propionic, or butyric acids during the same incubation period of 10 days. The loss from the acetic acid was 14 o/o, from propionic 11 o/o, and from butyric acid, 7 1 o/o, or slightly more than that due to the action of B. aerogenes capsulatus. Controls in distilled water lost from 2 to 3 o/o. The authors conclude that the erosive action of acids may be utilized clinically in dressing solutions. A 1 o/o acetic acid solution may hasten the disappearance of the sequestra and so destroy the focus of infection.

*The persistence of a sinus*, they believe, may often be due to the presence of organisms within the dead bone (whether in sequestra or in portions still attached to living bone) rather than to organisms in the soft tissues. So also the acute recrudescence of infection, or " flares ", after secondary operative procedures may be explained. Continual reinfection of the sinus tract must occur from the organisms in the sequestra at its bottom, either because of the erosion of the sequestrum or because of the outgrowth of bacteria from this nidus of infection. On the other hand, it by no means follows that the complete closing of a sinus indicates that the bone has become sterile. Growth was obtained from sequestra removed from cases in which the sinus had been closed for two weeks or longer. Hence the danger of infection from early intervention for bone grafting or nerve suture in which asepsis is a primary necessity. Many small portions of attached bone as well as sequestra may be centers of infection and therefore cause the failure of such operations. " Flares ", a term applied to the rise of temperature within 24 hours of sequestrectomy, and the associated inflammatory reaction about the wound, may probably be regarded as evidence of a temporary acceleration of the growth of organisms freed by the crushing of the bone and callus, and of the absorption of their toxic products. Such " flares " occurred in about 39 o/o of the 48 cases, and it was observed that 58 o/o of the " flares " were in cases of more than 120 days. It was also observed that a much higher percentage of the " flares "

(88.5 o/o as against 40.6) occurred in cases which, after the operation, were put on the Carrel-Dakin technique, than in those treated by dry dressings.

A much larger incidence of anaerobic gas-producing bacilli and spores was observed in the " flare " cases than in those with a normal reaction. This preponderance was greater in the new than in the old cases.

The authors conclude that anaerobic bacilli are probably active factors in the disintegration of sequestra within the wound and that anaerobic bacilli within the dead bone appear to be associated with the occurrence of " flares " after operations on bones. Since the sequestra are foci of infection, the object of treatment should be to remove the sequestra. These may be extracted by operation or their disappearance may be hastened by utilizing the solvent action of acid dressing solutions.

